# The Role of Xuefu Zhuyu Decoction in Prevention of Contrast-Induced Nephropathy after Percutaneous Coronary Intervention

**DOI:** 10.1155/2020/5419016

**Published:** 2020-04-27

**Authors:** Jingjing Zhao, Huahua Liu, Buyun Xu, Jiahao Peng, Yangbo Xing, Weiliang Tang, Fang Peng

**Affiliations:** ^1^Department of Cardiology, Shaoxing People's Hospital, Shaoxing Hospital of Zhejiang University, Shaoxing, Zhejiang 312000, China; ^2^Zhejiang University School of Medicine, Hangzhou, Zhejiang 310000, China; ^3^Loma Linda University School of Public Health, 24951 Circle Dr, Loma Linda, CA 92354, USA

## Abstract

**Objective:**

This study aimed to investigate the effect of Xuefu Zhuyu decoction on preventing contrast-induced nephropathy (CIN) after percutaneous coronary intervention (PCI).

**Methods:**

A total of 256 patients undergoing selective PCI for coronary artery disease were consecutively enrolled and randomly divided into two groups: Group A (*n* = 126) and Group B (*n* = 130). Before and after PCI, all patients routinely received antiplatelet aggregation therapy, antilipidemic therapy, and hydration therapy. Besides routine therapy, patients in Group B received Xuefu Zhuyu decoction from 3 days before PCI to 3 days after PCI. Serum creatinine (Scr), estimated glomerular filtration rate (eGFR), superoxide dismutase (SOD), and malondialdehyde (MDA) were measured, respectively, at baseline (72 h before PCI) and at 24, 48, and 72 h after PCI.

**Results:**

Compared with Group A, Group B presented a lower fluctuation of SCr and eGFR (*P* < 0.01). The incidence of CIN was less in Group B. According to the definition, CIN occurred in 5 patients (2.0%) in the intervention group and 5 (4.0%) in the control group (*P*=0.167). In terms of oxidative stress, Group B had a lower MDA (*P* < 0.05), but a higher SOD (*P* < 0.05).

**Conclusions:**

Compared with the control group, Xuefu Zhuyu decoction intervention therapy increased the level of SOD and reduced MDA. The Xuefu Zhuyu decoction intervention group presented a higher level of eGFR at 24, 48, and 72 h after PCI in patients with coronary heart disease and a lower level of Scr. The results are propitious to prove that Xuefu Zhuyu decoction might play an antioxidative stress role in the prevention of CIN after PCI.

## 1. Introduction

Contrast-induced nephropathy (CIN) is defined as an increase in serum creatinine beyond 44.2 *μ*mol/L (0.5 mg/dl) or 25% of the baseline within 72 h after contrast medium administration without other factors [1–4]. In recent years, more and more patients receive the therapy of percutaneous coronary intervention (PCI) [5]. A large amount of contrast media is needed during the procedure, and a dose-dependent relation between contrast media volume and CIN in patients undergoing angiography had been reported [6]. CIN is a common complication during the periprocedural period with an incidence rate ranging from 5% to 25% [7, 8]. Besides the high incidence of CIN [9, 10], it is associated with adverse outcomes, which has become one of the primary causes of secondary renal dysfunction. It was reported that the morbidity of CIN was 2%–30% after PCI [11]. Therefore, how to prevent and treat CIN is critical.

However, the pathogenesis of CIN is still unclear. There is no effective treatment for CIN nowadays. Several perioperative preventions have been developed [12, 13], including prophylactic intravenous hydration before and after operation, decreasing the dosages of contrast agents, and application of low- or iso-osmolar contrast medium. Nevertheless, the effect of hydration and Western pharmaceutical interventions on the prevention of CIN are still unsatisfied [14]. Given the limitations of Western medicine in preventing and treating CIN, traditional Chinese medicine might provide a complementary therapy for it. According to the previous studies, Xuefu Zhuyu decoction suppressed the oxidative stress reaction [15–17] which played an important role in the development of CIN [18–20] although the precise mechanisms of CIN were not completely elucidated. Therefore, we speculated that Xuefu Zhuyu decoction might contribute to reduce CIN after PCI probably through suppressing the oxidative stress reaction.

## 2. Methods

### 2.1. Study Population

Patients with coronary heart disease undergoing selective PCI in our hospital were continuously enrolled from June 2014 to May 2017. Exclusion criteria were as follows: (1) receiving other Chinese medicines one month before PCI; (2) allergic to the contrast medium; (3) using the contrast medium in the last week; (4) undergoing coronary artery bypass grafting; (5) malignant tumor; (6) acute and chronic infection; (7) emergency PCI; (8) severe cardiac dysfunction (left ventricular ejection fraction <30%); (9) severe hepatic and renal dysfunction (estimated glomerular filtration rate <30 ml·min^−1^·1.73^−2^ or creatinine clearance rate <30 ml/min or ALT/AST >3 times the normal value); and (10) antithrombotic intolerance. The protocol was approved by the Ethics Committee of Shaoxing People's Hospital (the Ethics Committee of Shaoxing People's Hospital consists of 16 members, including one chairman and two vice-chairmen), and all participants gave written informed consent.

### 2.2. Study Methods

We planned to recruit 300 patients, but we actually recruited 256 patients. Patients were randomly 1 : 1 divided into two groups (control group and intervention group) by using table of random number. After treatment plan communications with patients, a total of 256 patients undergoing selective PCI for coronary artery disease were divided into two groups: the control group (*n* = 126) and the intervention group (*n* = 130). Before PCI, all patients were given aspirin (100 mg/day *∗* 7 days, orally), clopidogrel (loading dose of 300 mg/day), and atorvastatin (20 mg/night). After PCI, all patients continued to take aspirin and clopidogrel (75 mg/day) for at least 12 months and were injected subcutaneously low molecular weight heparin 0.4 ml/day for 3 days. Atorvastatin was continued if no contraindication existed.

All patients received intravenous hydration with isotonic saline (1 ml/kg/h, 0.9% sodium chloride for 8 h both before and after the use of angiography). The hydration rate was reduced appropriately for patients with left ventricular ejection fraction (LVEF) <50%. Patients in the intervention group (Group B) received Xuefu Zhuyu decoction from 3 days before PCI to 3 days after PCI, and the control group (Group A) did not.

Xuefu Zhuyu decoction is provided by the preparation room of Shaoxing People's Hospital. The formula of Xuefu Zhuyu decoction contains peach kernel (13.7 g), safflower (10.3 g), *Angelica* (9 g), raw rehmannia (9 g), sichuan dome (5 g), red peony (6 g), *Achyranthes bidentata* (9 g), *Platycodon* (5 g), *Bupleurum* (3 g), fructus aurantii (6 g), and liquorice (3 g). The decocting methods are (1) soaking in warm water for 1 hour, (2) decocting in gentle fire for 45 minutes, (3) extracting the juice and then decocting the two juices, (4) decocting about 400 ml twice, and (5) taking two times in the morning and evening after mixing, at 1 dose a day.

The study was a randomised, controlled, single-blinded clinical trial. The physicians performing the angiographic procedure were blinded to the assignment group. The same nonionic, low-osmolar contrast medium (Iobitridol injection, France GUERBET) was used in all cases.

Serum creatinine (Scr), superoxide dismutase (SOD), and malondialdehyde (MDA) were measured at baseline (72 h before PCI) and at 24, 48, and 72 h after PCI by collecting fasting venous blood. The estimated glomerular filtration rate (eGFR) was calculated according to the modified MDRD formula which is suitable for Chinese: eGFR (ml·min^−1^·1.73^−2^) = 175 × SCr (mg/dl) −1.154 × age − 0.203 (×0.742, if female). All tests were performed in our center laboratory blinded to assignment.

### 2.3. Definition of CIN

CIN is defined as an increase in serum creatinine beyond 44.2 *μ*mol/L (0.5 mg/dl) or 25% of the baseline within 72 h after contrast medium administration without other factors.

### 2.4. Statistical Analysis

Continuous variables were indicated as mean ± SD and categorical variables as percentages. The differences for continuous variables between the two groups were estimated through the independent sample *t*-test. Holm–Sidak test was used to perform multiple comparisons. Chi-square was applied to compare categorical variables between groups. Univariate and multivariate linear regression analyses were performed to investigate the factors associated with renal function. All data were analyzed through SPSS 24. A *P* value less than 0.05 was considered significant (2 sided).

## 3. Results

### 3.1. Patient Population

A total of 256 patients with coronary heart disease undergoing selective PCI at our hospital were continuously enrolled from June 2014 to May 2017. After random allocation, 126 patients only received routine treatments as mentioned above (Group A). The remaining 130 patients were classified as Group B and received intervention treatment of Xuefu Zhuyu decoction in addition to routine treatments.

Demographic characteristics and clinical, biochemical, and procedural variables are presented in Table 1. The mean age of population was 59 years. 149 patients were male. The mean LVEF was 59%. 23 patients (9%) had previous MI, and 26 patients (10%) presented multivessel disease. Mean eGFR at baseline was 82.47 ml/min *∗* 1.73 m^2^. Mean volume of the contrast medium was 31.98 ml. There were no significant differences between the two groups in sex, LVEF, hypertension, clinical presentation, and laboratory variables at baseline (*P* > 0.05).

### 3.2. Analysis of Renal Function

**Figure 1:**
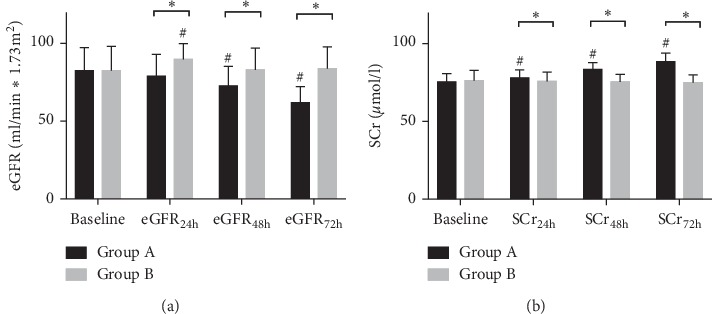
(a) eGFR before and after PCI. Group (A): control group and Group (B): intervention group; ^#^compared with baseline, adjusted *P* value<0.0001; ^*∗*^adjusted *P* value <0.0001. (b) SCr before and after PCI. Group (A): control group and Group (B): intervention group; ^#^compared with baseline, adjusted *P* value <0.01; ^*∗*^adjusted *P* value <0.01. eGFR: estimated glomerular filtration rate; SCr: serum creatinine.

In Group A, compared with baseline, SCr increased significantly after PCI (SCr_24h_ vs SCr_baseline_: 78.1 *µ*mol/l vs 75.6 *µ*mol/l, *P*=0.0014; SCr_48h_ vs SCr_baseline_: 83.6 *µ*mol/l vs 75.6 *µ*mol/l, *P* < 0.0001; SCr_72h_ vs SCr_baseline_: 88.5 *µ*mol/l vs 75.6 *µ*mol/l, *P* < 0.0001) and accordingly eGFR reduced significantly in Group A (eGFR_24h_ vs eGFR_baseline_: 78.9 ml/min *∗* 1.73 m^2^ vs 82.6 ml/min *∗* 1.73 m^2^, *P*=0.1554; eGFR_48h_ vs eGFR_baseline_: 72.8 ml/min *∗* 1.73 m^2^ vs 82.6 ml/min *∗* 1.73 m^2^, *P* < 0.0001; eGFR_72h_ vs eGFR_baseline_: 61.9 ml/min *∗* 1.73 m^2^ vs 82.6 ml/min *∗* 1.73 m^2^, *P* < 0.0001). However, no significant difference was identified between baseline and postprocedural SCr and eGFR in Group B, except for eGFR at 24 h which was significantly higher than baseline (eGFR_24h_ vs eGFR_baseline_: 89.8 ml/min *∗* 1.73 m^2^ vs 82.4 ml/min *∗* 1.73 m^2^, *P* < 0.0001). When compared between the two groups, Group B presented a higher level of eGFR at 24, 48, and 72 h after PCI (*P* < 0.0001, respectively), and accordingly the level of SCr was significantly lower in Group B (*P* < 0.01, respectively). The results are summarized in Figure 1. According to definition, CIN occurred in 5 patients (2.0%) in the intervention group and 5 (4.0%) in the control group (*P*=0.167).

### 3.3. Comparison of Markers of Oxidative Stress

**Figure 2:**
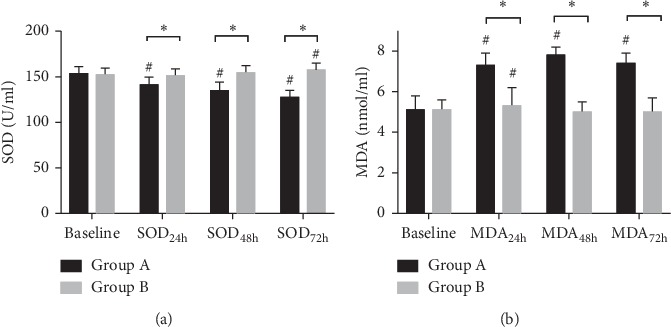
(a) SOD at baseline and after PCI. Group (A): control group; Group (B): intervention group; ^#^compared with baseline, adjusted *P* value<0.0001; ^*∗*^adjusted *P* value <0.0001. (b) MDA at baseline and after PCI. Group (A): control group; Group (B): intervention group; ^#^compared with baseline, adjusted *P* value<0.05; ^*∗*^adjusted *P* value <0.0001.

In Group A, compared with baseline, SOD reduced significantly after PCI (SOD_24h_ vs SOD_baseline_: 141.2 U/ml vs 153.4 U/ml, *P* < 0.0001; SOD_48h_ vs SOD_baseline_: 134.7 U/ml vs 153.4 U/ml, *P* < 0.0001; SOD_72h_ vs SOD_baseline_: 127.6 U/ml vs 153.4 U/ml, *P* < 0.0001) and MDA increased significantly (MDA_24h_ vs MDA_baseline_: 7.3 nmol/ml vs 5.1 nmol/ml, *P* < 0.0001; MDA_48h_ vs MDA_baseline_: 7.8 nmol/ml vs 5.1 nmol/ml, *P* < 0.0001; MDA_72h_ vs MDA_baseline_: 7.4 nmol/ml vs 5.1 nmol/ml, *P* < 0.0001). However, no significant difference was identified between baseline and postprocedural SOD and MDA in Group B, except for SOD at 72 h which was significantly higher than baseline (SOD_72h_ vs SOD_baseline_: 157.6 U/ml vs 152.3 U/ml, *P* < 0.0001) and MDA at 24 h was significantly higher than baseline (MDA_24h_ vs MDA_baseline_: 5.3 nmol/ml vs 5.1 nmol/ml, *P*=0.0461). When compared between the two groups, Group B presented a higher level of SOD at 24, 48, and 72 h after PCI (*P* < 0.0001, respectively), and accordingly the level of MDA was significantly lower in Group B (*P* < 0.0001, respectively). The results are summarized in Figure 2.

### 3.4. Relationship between Renal Function and Oxidative Stress

The term “deta” represents the change value of an indicator. Significant linear relation between detaSCr and detaSOD (*R*^2^ = 0.7843, *P* < 0.0001) was indicated in linear regression analysis. Similarly, detaSCr was linearly correlated to detaMDA (*R*^2^ = 0.7504, *P* < 0.0001). Moreover, detaGFR had significant linear correlation with detaSOD (*R*^2^ = 0.8792, *P* < 0.0001), and detaGFR was linearly related to detaMDA (*R*^2^ = 0.8938, *P* < 0.0001).

### 3.5. Risk Factors Associated with eGFR, SCr, SOD, and MDA

To explore the factor influencing the results of eGFR, SCr, SOD, and MDA at 72 h, univariate linear regression analyses were performed.  Table 2 presents the factors with *P* value <0.05. Intervention with Xuefu Zhuyu decoction was associated with all measurement indexes, contributing to higher eGFR (*P* < 0.01), lower SCr (*P* < 0.01), higher SOD (*P* < 0.01), and lower MDA (*P* < 0.01).

Stepwise multivariate regression analysis was used to explore independent factors associated with measurement indexes. Potential confounders based on a known relevant clinical factor and factors with a *P* value less 0.1 during univariate analyses were tested in the multivariate model. The results are presented in Table 3. In the multivariate regression analysis, intervention with Xuefu Zhuyu decoction was still an independent factor predicting higher eGFR (*P* < 0.01), lower SCr (*P* < 0.01), higher SOD (*P* < 0.01), and lower MDA (*P* < 0.01).

When adding detaSOD and detaMDA into independent variables, we observed that intervention measures still significantly contributed to detaSCr/eGFR (*P* < 0.01) in stepwise multivariate regression analysis.

## 4. Discussion

To our knowledge, the present study was the first study evaluating the role of Xuefu Zhuyu decoction in prevention of CIN. The main findings were as follows: (i) Xuefu Zhuyu decoction was associated with less SCr and higher eGFR after PCI; (ii) Xuefu Zhuyu decoction contributed to higher SOD and lower MDA, which are the markers of oxidative stress that might play an important role in CIN; (iii) oxidative stress played an important role in the pathogenesis of CIN.

Multiple mechanisms may be involved in the development of CIN, including decreased renal blood flow, renal tubular microembolization, apoptosis, oxidative stress, and contrast agents' direct toxicity to renal tubular epithelial cells [21–23]. It had been found that the mechanism of CIN is mainly due to the direct toxic effect of contrast agents on renal tubular epithelial cells through oxygen-free radicals and inflammatory response factors [24, 25]. Gong et al. [26] had shown that the occurrence of CIN could be prevented by inhibiting oxidative stress. Contrast medium significantly attenuated renal SOD and GSH (glutathione) levels and increased MDA levels. They found that contrast medium-induced indicators of oxidative stress in the kidney and the CIN-induced changes could be blocked through their pretreatment. Buyuklu et al. [27] had found that the incidence of CIN could be reduced via inhibiting oxidative stress. They found a significant increase in MDA and a significant decrease in GSH and SOD levels in the CIN group compared with the control group. Ozturk et al. [28] also had indicated that the biochemical and histopathological deleterious effects of CIN might be alleviated via enhancing total antioxidant capacity and decreasing oxidative stress.

At present, hydration therapy is generally recognized as the routine measure to prevent CIN. Although adequate hydration therapy reduces the concentration and hyperosmotic state of the contrast media [29], the incidence of CIN after PCI is still high [30] and hydration therapy cannot decrease the production of oxygen-free radicals. As the contrast media can cause a series of oxidative stress reaction, several compounds with properties of antioxidant stress such as sodium bicarbonate, N-acetylcysteine, ascorbic acid, and statins have been investigated [31]. However, the mechanism of sodium bicarbonate was primarily inferred from animal experiment studies. N-Acetylcysteine was likely to only decrease SCr concentration without really preventing CIN. The paradoxical results of ascorbic acid had been found, and there were no benefits when ascorbic acid was used intravenously in patients undergoing cardiac catheterization with renal dysfunction. The effects of statins on CIN were indeterminate in recent studies, most research studies about the effect of statins on CIN were performed through intra-arterial administration of contrast agents, and the use of statins in radiology patients should be cautious because of insufficient evidence [31]. Xuefu Zhuyu decoction, a Chinese traditional medicine prescription for improving blood circulation and alleviating blood stasis [32], has been widely used for a long time clinically in China. Meng et al. [15] found that Xuefu Zhuyu decoction decreased the levels of MDA, increased the levels of SOD in serum or in heart in sepsis rats, it had the function of alleviating myocardial injury, and had a certain antioxidation effect; their findings suggested that pretreatment with Xuefu Zhuyu decoction put a protective effect in the myocardium of septic rats through inhibiting myocardial cell apoptosis and antioxidation. Fan et al. [16] collected serum and isolated tissue fluid, cytosols, and microsomes from liver tissues by centrifugation according to the standard procedure, when male SD rats were treated with Xuefu Zhuyu decoction at different dosages per day for 15 days. Their findings suggested Xuefu Zhuyu decoction induced the activities of SOD and increased GSH (glutathione) level in the liver of rats, which indicated that Xuefu Zhuyu decoction might have detoxification and antioxidant functions. In our study, we found that significant linear relation existed between renal function and markers of oxidative stress. The results suggested oxidative stress played an important role in CIN, which was consistent with previous studies. In addition, we also demonstrated that Xuefu Zhuyu decoction was associated with SOD and MDA which were generally accepted as markers of oxidative stress. Accordingly, Xuefu Zhuyu decoction also contributed to less SCr and higher eGFR in patients receiving PCI, compared with those who did not receive Xuefu Zhuyu decoction. The results indicated that Xuefu Zhuyu decoction might prevent CIN after PCI mainly through antioxidative stress effect. Interestingly, after adjusting for SOD and MDA, Xuefu Zhuyu decoction still independently influenced renal function, which suggested that oxidative stress was not the only mechanism underlying the prevention of CIN by Xuefu Zhuyu decoction, and further studies need to be carried out.

Xuefu Zhuyu decoction is a well-known traditional Chinese medicine for treating cardiovascular diseases, and the findings of our study suggested its effects on antioxidative stress and contributions to prevent CIN after PCI; the findings might improve prognosis of patients receiving PCI. The specific mechanism of CIN is not fully understood, and the results of our study might provide a theoretical and practical basis for the pathogenesis of CIN, a possible new perspective for prevention and treatment of CIN, and a clearer picture for subsequent research on the treatment of CIN. Then, if our research results could be put into practice, the potential possibility of long-term renal insufficiency would greatly be decreased, the hospitalization time and cost of patients might remarkably be reduced, and the postoperative quality of life of patients would well be improved.

### 4.1. Study Limitations

The current study presented several limitations. Firstly, due to small sample size and rare incidence of CIN in our study, the current study was underpowered to demonstrate that Xuefu Zhuyu decoction could reduce CIN. However, we found that Xuefu Zhuyu decoction was associated with higher level of eGFR and lower SCr after PCI. The results may provide a theoretical basis to conduct larger studies to explore the relationship between Xuefu Zhuyu decoction and CIN. Secondly, the conclusion of this study was based only on the results of clinical tests, and no in-depth study of histopathological and molecular mechanism changes of CIN before and after the intervention was done. Thirdly, this study only observed one of the mechanisms of Xuefu Zhuyu decoction on renal protection, and deeper antioxidant stress and other mechanisms of CIN remain to be explored.

## 5. Conclusion

Compared with the control group, Xuefu Zhuyu decoction intervention therapy increased the level of SOD and reduced MDA. The Xuefu Zhuyu decoction intervention group presented a higher level of eGFR at 24 h, 48 h, and 72 h after PCI in patients with coronary heart disease, and the level of SCr was significantly lower. The results are propitious to prove that Xuefu Zhuyu decoction might play an antioxidative stress role in the prevention of CIN after PCI.

## Figures and Tables

**Table 1:** General characteristics and relative clinical and biochemical variables.

	Group A (*n* = 126)	Group B (*n* = 130)	*P* value
Age (years)	60 ± 9	59 ± 9	0.52
Male (*n*)	71 (56%)	78 (60%)	0.55
Height (meter)	1.64 ± 0.09	1.63 ± 0.09	0.81
Weight (kg)	67 ± 10	68 ± 10	0.41
LVEF (%)	60 ± 10	58 ± 12	0.31
HF (*n*)	24 (19%)	28 (22%)	0.62
Hypertension (*n*)	68 (54%)	71 (55%)	0.92
Diabetes (*n*)	24 (18%)	25 (20%)	0.97
Smoking (*n*)	47 (37%)	41 (32%)	0.33

*Clinical presents*			
Previous MI (*n*)	12 (10%)	11 (8%)	0.77
Multivessel disease	12 (10%)	14 (11%)	0.74
Contrast volume (ml)	31.39 ± 9.86	32.55 ± 9.67	0.35

*Lab variables at baseline*			
eGFR (ml/min *∗* 1.73 m^2^)	82.58 ± 14.80	82.37 ± 15.56	0.91
SCr (*µ*mol/l)	75.62 ± 5.29	76.34 ± 6.60	0.34
SOD (U/ml)	153.36 ± 7.82	152.27 ± 7.30	0.25
MDA (nmol/ml)	5.11 ± 0.73	5.06 ± 0.51	0.53

Group A: control group; Group B: intervention group; LVEF: left ventricular ejection fraction; HF: heart failure; MI: myocardial infarction; eGFR: estimated glomerular filtration rate; SCr: serum creatinine; SOD: superoxide dismutase; MDA: malondialdehyde; LVEF:left ventricular ejection fraction; HF:heart failure.

**Table 2:** Results of univariate linear regression (95% confidence interval of regression coefficient).

Variables	Intervention	Male	Smoking	HF	Weight	Height	Age
eGFR_72h_	21.81 (18.74, 24.87)	21.78 (18.64, 24.92)	11.41 (7.35, 15.47)	−5.14 (−10.17, −1.05)	0.71 (0.51, 0.90)	84.43 (63.54, 105.33)	−0.61 (−0.83, −0.39)
SCr_72h_	−13.60 (−14.89, −12.31)	—	—	—	—	—	0.35 (0.23, 0.46)
SOD_72h_	30.03 (28.21, 31.86)	12.45 (8.55, 16.35)	5.08 (0.77, 9.39)	—	0.43 (0.22, 0.64)	43.95 (20.91, 67.00)	−0.41 (−0.64, −0.18)
MDA_72h_	−2.50 (−2.64, −2.35)	−0.98 (−1.30, −0.66)	—	—	−0.03 (−0.05, −0.02)	−3.38 (−5.28, −1.48)	0.03 (0.02, 0.05)

eGFR_72h_: eGFR at 72 h after PCI; SCr_72h_: SCr at 72 h after PCI; SOD_72h_: SOD at 72 h after PCI; MDA_72h_: MDA at 72 h after PCI. ^#^Male vs female 60.34 (eGFR); 165.55 (SOD); 4.48 (MDA). ^*∗*^Per 10 cm 8.44 (eGFR); 4.40 (SOD); −0.34 (MDA).

**Table 3:** Results of multivariate linear regression (95% confidence interval of regression coefficient).

Variables	Intervention	Male	Smoking	Weight	Age
eGFR_72h_	20.76 (19.54, 21.98)	18.79 (17.10, 20.49)	1.96 (0.44, 3.48)	0.09 (0.02, 0.17)	−0.53 (−0.60, −0.46)
SCr_72h_	−13.37 (−14.47, −12.27)	—	—	—	0.32 (0.25, 0.38)
SOD_72h_	29.39 (28.42, 30.35)	11.21 (10.24, 12.19)	—	—	−0.33 (−0.38, −0.28)
MDA_72h_	−2.44 (−2.52, −2.37)	−0.88 (−0.95, −0.80)	—	—	0.03 (0.02, 0.03)

eGFR_72h_: eGFR at 72 h after PCI; SCr_72h_: SCr at 72 h after PCI; SOD_72h_: SOD at 72 h after PCI; MDA_72h_: MDA at 72 h after PCI. Adjusting for hypertension, diabetes, previous myocardial infarction, multivessel disease, contrast volume, and LVEF.

## Data Availability

The data used to support the study are available from the corresponding authors Pang Peng (email: sxrmyypf@126.com) and Weiliang Tang (email: twl-sxyz@163.com).
